# Is it all about knowledge? A survey of attitudes toward ADHD among German pediatricians

**DOI:** 10.1007/s10389-022-01758-4

**Published:** 2022-09-23

**Authors:** Marie E. Fechner, Yuliya Mazheika, Folkert Fehr, Ronny Jung, Peter Borusiak

**Affiliations:** 1 Witten/Herdecke University, Witten, Germany; 2 Wagener-Foundation for Social Pediatrics, Emsdetten, Germany; 3 Pediatric Practice, Sinsheim, Germany; 4 Pediatric Practice, Roth, Germany; 5 Kinderneurologisches Zentrum KiNZ, LVR-Klinik, Waldenburger Ring 46, D-53119 Bonn, Germany

**Keywords:** Attention-deficit/hyperactivity disorder, ADHD, Treatment, Attitude, Child, Pediatrician

## Abstract

**Aim:**

Sometimes treatment is not necessarily according to guidelines, which is partly due to attitudes rather than lack of knowledge. In Germany, regional differences concerning prevalence rates of ADHD exist without valid explanation. We contribute with our data to the question of attitudes toward ADHD among pediatricians in Germany.

**Method:**

A specially designed questionnaire based on the Knowledge of Attention Deficit Disorders Scale and previous surveys was sent to pediatricians. In addition to descriptive statistics, we performed factor analysis and multiple linear regression analysis.

**Results:**

The vast majority (85.5%) of 581 respondents have a positive attitude toward ADHD, i.e., ADHD exists and should be treated appropriately. Physicians with positive attitudes were older and more often had a neuropediatric specialization.

**Conclusion:**

Most of the pediatricians surveyed in Germany assess ADHD and treatment in line with medical guidelines and treat as part of multimodal therapy.

**Supplementary Information:**

The online version contains supplementary material available at 10.1007/s10389-022-01758-4.

## Introduction

Attention-deficit hyperactivity disorder (ADHD) is one of the most common behavioral disorders (Göbel et al. 2018) with a worldwide prevalence of approximately 5.3% without much variation over time. The prevalence in Germany is largely consistent with these figures (Bachmann et al. 2017; Göbel et al. 2018). Variability in ADHD prevalence estimates is mostly explained by methodological differences of the studies (Polanczyk et al. 2014). Consequences of this disease are sometimes serious comprising substantial individual, academic, and social functioning.

Different health care systems exist worldwide with distinctive approaches to specialized care in child and adolescent mental health services. In some countries, general practitioners have a gatekeeper function; in others, the specialist can be consulted directly. Many publications from different countries point to under-diagnosis and under-treatment as not all young people with ADHD had seen a relevant health specialist (Sayal et al. 2006; Tremmery et al. 2007).

International guidelines and treatment recommendations consistently favor three different treatment approaches comprising psychoeducation, behavioral interventions, and medication (NICE guideline 2018; Wolraich et al. 2019). Generally, using an evidence-based methodology stimulant medication has proven to be the most effective approach. In addition, some other nonstimulant medications are used, however, based on smaller effect sizes. Despite concurring recommendations of the guidelines, only half of the people meeting diagnostic criteria for ADHD in the USA will ever make treatment contact (Power et al. 2005). Additionally, treatment initiation and adherence are not only dependent on contact with health care specialists and guidelines but also from individual attitudinal factors of patients, their parents, as well as teachers and last but not least physicians. There are several studies pointing to different parent-related attitudinal factors influencing treatment of children with ADHD. These factors include fear of stigma (Gulliver et al. 2010; DosReis et al. 2010; Pescosolido et al. 2008), self-conception (Jiang et al. 2014) as well as conception of cause of ADHD-typical behavior (Jiang et al. 2014). Just recently, a study focused on factors associated with treatment attitudes and information-seeking behavior by parents identifying knowledge and understanding of ADHD and resistance to stigma as related to positive attitudes toward ADHD treatment (Taylor and Antshel 2021). Most of those studies focus on parents, teachers or children themselves (Rodrigo et al. 2011; Gilmore 2010; Canu and Mancil 2012) and only a few include health care professionals and especially pediatricians (Ghanizadeh and Zarei 2010; Adamis et al. 2018).

Tatlow-Golden published a review of knowledge of general practitioners as primary gatekeepers (Tatlow-Golden et al. 2016). However, services are different in distinctive countries (Hinshaw et al. 2011) with different function of general practitioners, pediatricians, and specialized services.

## Objective

Conceptual considerations and social attitudes play a role, especially in the case of psychiatric illnesses or mental health problems. In Germany, very large regional differences have also been found with regard to the prevalence of ADHD, which have persisted even in small-scale regional observations and over longer time courses. In some regions of Germany, the prevalence is less than half the expected value, while in others it is more than twice as high (Grobe 2017; Akmatov et al. 2018). Overall, prevalence rates range from 1.6% to 9.7% (with a mean of 4.3%). A similar range is seen for drug treatment with stimulants (Akmatov et al. 2018). The differences are manifold and not only visible in a comparison between federal states or between urban and rural regions but also in small-scale comparisons. Part of the variation can be explained by this and by the density of physicians. However, a large part of the variation cannot be explained with the data available so far. No plausible and completely satisfactory explanation has yet been found for the variations in ADHD prevalence and methylphenidate prescription rates within Germany. Various factors can influence diagnoses and their frequency. In addition to the different density of specialists and possible misdiagnoses, another factor may be the attitude toward the disease – an aspect that has not yet been systematically investigated. This study is intended to contribute to this.

A definition of “attitude” is complex and is handled variably in different contexts. A depiction is beyond the scope of this article. In previous publications, various terms have been used mostly synonymously: e.g., “understanding” and “perception” (Salt et al. 2005), “conceptions” (Taylor and Antshel 2021), “conceptualization” (Adamis et al. 2018). All authors predominantly use the term “attitude.” In our study, attitude refers to the clinician’s readiness, resulting from experience, to respond in a certain way to a situation or the ADHD clinical picture. This can be expressed in cognitive (assumptions and beliefs), affective (feelings and emotions), and behavioral terms. For a somewhat more detailed discussion of the various influencing factors in ADHD, see the review paper (Tatlow-Golden et al. 2016). Attitude research clarifies the connections between attitudes, behavior, and action. ADHD is a disorder resulting from a complex interaction between neurobiological, genetic, psychological, and social factors. Since there are still no biomarkers for ADHD, the diagnostic concept is based solely on behavioral phenotype. In addition, although the scientific community agrees that ADHD is a disorder based on neurobiological factors, not all physicians hold this opinion. The following questions will be investigated with our study:

1. What are the attitudes of pediatricians toward ADHD and possible drug therapy with stimulants in Germany?
2. Are there regional differences in the attitudes of pediatricians toward ADHD and possible drug therapy with stimulants in Germany?
3. If so, can correlations between these attitudes and prevalence data and/or methylphenidate prescription rates be shown?

## Methods

### Participants and procedures

In Germany there is a comprehensive system of pediatricians in private practice caring for children that is by large means financed by a system of statuary health insurance companies. Some of pediatricians have additional specialization as neuropediatricians, others a (non-formal) specialization with focus on developmental problems. Also, a pediatric working group on children with ADHD exists. Additionally, there are approximately 1100 residential child and youth psychiatrists.

Participants of this cross-sectional descriptive study were recruited by the mailing list of members of the Association of Child and Adolescent Physicians in Germany (Berufsverband der Kinder- und Jugendärzte, BVKJ). This association represents the interests of its approximately 12,000 members in rights, organizational, social, and political matters. Most of the members are working as residential pediatricians. The survey was conducted between November 1, 2020 until December 11, 2020, and programmed with the online questionnaire tool “SoSci Survey” (www.soscisurvey.de). After completion of the questionnaire, the link to the survey was sent by e-mail to those members of the BVKJ who have provided an e-mail address and have given the BVKJ their consent to receive information or inquiries by e-mail (*n* = 5531).

### Questionnaires

**Fig. 1:**
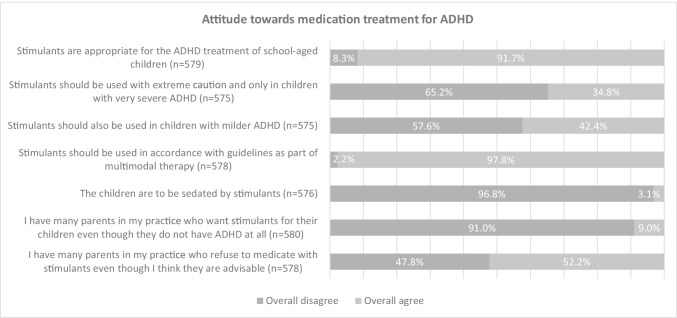
Attitudes of pediatricians toward stimulant treatment for ADHD in Germany

The Knowledge of Attention Deficit Disorders Scale (KADDS) serves as the basis for the survey. The KADDS is a validated instrument for the assessment of knowledge about symptoms, diagnostics, causes and interventions in ADHD, which is also used in international studies (Sciutto et al. 2000) and for which a German version also exists (Schmiedeler 2013). Based on the KADDS, we created a questionnaire adapted to the research question. Since parts of the KADDS focus primarily on knowledge, the questions on attitudes were supplemented, also drawing on questions already used in other studies (e.g., Adamis et al. 2018; Ghanizadeh and Zarei 2010; Fitzgerald and McNicholas 2014; Shaw et al. 2002). The questionnaire was also adapted to the German health care system and the survey population, and additional demographic questions relevant to our study were added. The complete questionnaire is added to this publication as supplementary material. In this publication, we have focused on the following topics of the survey:

- *Demographic data, qualification and place/area of work*: This section (6 Questions) dealt with demographic details of the participants: age, gender, specialist for pediatrics and adolescent medicine, professional years as a pediatrician, focus on neuropediatrics, work area and place (Table 1).
- *Personal opinion concerning prevalence of ADHD in Germany*: We examined this topic with the question “How do you assess the prevalence of ADHD in Germany?” Answers were divided into five categories, including “ADHD is overdiagnosed,” “The diagnosis is mostly correct in relation to the prevalence,” “ADHD is underdiagnosed,” “ADHD is sometimes underdiagnosed and sometimes overdiagnosed,” and “no answer.”
- *Degree of specialization*: This part was examined using two questions. The first question “How do you diagnose children with ADHD?” had five response categories: “In my practice there are no children with ADHD,” “I do not perform diagnostics in my practice, but I accept patients diagnosed with ADHD,” “I actively refer patients with suspected ADHD to a cooperation partner,” “I carry out the diagnostics myself in my practice according to my own specifications,” and “I carry out the diagnostics in my practice myself according to the guidelines.” For the second question “How do you treat children with ADHD?” we established the following response categories: “I do not treat children and adolescents with ADHD,” “I treat children and adolescents with ADHD, but I do not write prescriptions,” “I treat children and adolescents with ADHD, issue prescriptions if the indication was made by other cooperation partners,” “I treat children and adolescents with ADHD independently and also write prescriptions myself,” and “other.”
- *Attitudes toward ADHD*: This section included 12 statements about beliefs, attitudes, and the etiology of ADHD (Table 2) and was rated on a 4-point Likert scale ranging from 1 = “strongly disagree” to 4 = “strongly agree.”
- *Attitudes toward medication treatment for ADHD:* This section contained seven statements about attitudes toward the need for medicament treatment for ADHS and parents’ role in the decision (Fig. 1). The questions were rated analogously to questions on “ADHD attitudes” on a 4-point Likert scale from 1 = “strongly disagree” to 4 = “strongly agree.”

**Table 1:** Characteristics of the study population

Characteristics		Study population n (*N* = 581)	Percent (%)
Sex	Female	312	53.7
	Male	261	44.9
	Diverse	0	0
	I do not wish to specify	6	1.0
	Missing values	2	0.3
Age groups	26–35 years	11	1.9
	36–45 years	121	20.8
	46–55 years	192	33.0
	56–65 years	219	37.7
	>66 years	31	5.3
	Missing values	7	1.2
Pediatrician	Yes	555	95.5
	No	10	1.7
	Missing values	16	2.8
Professional years as a pediatrician	< 5 years	42	7.2
	6–10 years	73	12.6
	11–15 years	76	13.1
	16–20 years	100	17.2
	21–25 years	100	17.2
	26–30 years	85	14.6
	31–35 years	55	9.5
	36–40 years	15	2.6
	>40 years	6	1.0
	Missing values	29	5.0
Neuropediatrician	Yes	76	13.1
	No	496	85.4
	Missing values	9	1.5
Area of work	Private practice (single)	223	38.4
	Private practice (group)	282	48.5
	Hospital	4	0.7
	Social pediatric center	67	11.5
	Public health service	1	0.2
	Other	2	0.3
	Missing values	2	0.3
All percentages may not total 100% due to rounding

**Table 2:** Exploratory factor analysis results of attitudes toward ADHD

Statements	Counts (%)				Two-factor loadings	
	strongly disagree	rather disagree	somewhat agree	strongly agree	Factor 1 (positive attitudes)	Factor 2 (negative attitudes)
ADHD is a clearly defined psychiatric disorder	18 (3.1)	95 (16.4)	307 (53.0)	159 (27.5)	.626	
ADHD is a new, ‘fashionable’ disorder	223 (38.8)	222 (38.6)	110 (19.1)	20 (3.5)		.498
ADHD is society’s excuse for badly behaved children	307 (53.0)	177 (30.6)	80 (13.8)	15 (2.6)		.701
An ADHD diagnosis is helpful for a child	16 (2.8)	68 (11.8)	322 (56.0)	169 (29.4)	.657	
An ADHD diagnosis is stigmatising for a child	61 (10.6)	291 (50.4)	204 (35.4)	21 (3.6)	−.474	
Children with ADHD misbehave because they do not want to follow the rules	405 (69.9)	142 (24.5)	29 (5.0)	3 (0.5)		.600
Parents seek ADHD diagnosis as an excuse for their child’s bad behaviour	241 (41.8)	252 (43.7)	73 (12.7)	11 (1.9)		.774
An ADHD diagnosis relieves families from stress and supports problem-solving	15 (2.6)	40 (6.9)	305 (52.7)	219 (37.8)	.627	
The etiology of ADHD lies in a predominantly genetically caused cerebral developmental disorder	31 (5.4)	126 (21.8)	273 (47.2)	148 (25.6)	.575	
Chaotic and dysfunctional family is the etiology of ADHD	159 (27.5)	284 (49.1)	121 (20.9)	14 (2.4)		.624
ADHD can be caused by poor parenting practices	323 (55.6)	206 (35.5)	46 (7.9)	6 (1.0)		.653
I feel confident in dealing with patients with ADHD	22 (3.8)	98 (16.9)	302 (52.2)	157 (27.1)	.502	
Cronbach’s Alpha					.687	.761

The first draft questionnaire was reviewed for errors and gaps in a total of three rounds of testing, with five pediatricians participating in the first round of review and 12 physicians participating in each of the other two rounds, six of whom had a more extensive specialization or focus on ADHD.

### Statistical analysis

Data analyses were performed with SPSS V. 26. Descriptive statistics (absolute and relative frequencies) were computed to describe demographic data, qualification and place/area of work, opinions on the appropriateness prevalence of ADHD, degree of specialization, attitudes toward ADHD and medication treatment for ADHD of the total sample.

The structure of the instruments surveying attitudes toward ADHD (12 statements) and attitudes toward ADHD medication (7 statements) was examined using two separate exploratory factor analyses. We used to force two-way factor solution analysis for each of these question blocks using principal component analysis extraction with varimax rotation and Kaiser normalization to obtain interpretable factors (“positive” and “negative” analogous to Adamis et al. 2018,1).

Finally, a multiple linear regression was conducted to determine which demographic characteristics and qualification were associated with positive and negative attitudes of pediatricians toward ADHD in Germany. Significance level was set to *p* < .05 (two-tailed).

### Ethical aspects

The study authors respected and abided by the legal version of the Declaration of Helsinki, published by the World Medical Association (WMA). Electronic informed consent was obtained before the survey began. Consent was given by actively ticking the consent checkbox. Study fully respected German data protection regulations.

## Results

Of a total of 5531 invited pediatricians, 627 (11%) responded to the questionnaire. In a first step, we checked the data sets for completeness. Those responses where the survey was terminated immediately (*n* = 6), after informed consent (*n* = 7), or after demographic data entry (*n* = 21) were not considered for analysis. In addition, there were 12 records with more than one missing response concerning the attitudes that we also excluded. Here, however, there was no clustering as to the respective missing responses. A total of 46 data records could therefore not be considered for the evaluation. Thus, after data cleaning, 581 questionnaires were available for further analysis.

Demographics and other sample descriptions are presented in Table 1.

### Personal opinion on the prevalence of ADHD

There was considerable variability in responses regarding personal opinion of current frequency of ADHD in Germany. Almost half of respondents (271/578) rated ADHD as sometimes underdiagnosed and sometimes overdiagnosed, while 9.1% thought that ADHD was underdiagnosed, 19.6% overdiagnosed, and 20.8% thought the rate of diagnosis was appropriate. Of the 578 participants, 19 answered the question with “no answer.”

### Degree of specialization

Of respondents 64% (372/580) reported referring patients to a collaborative partner when ADHD was suspected, 28.2% performed the diagnosis in the practice itself according to the guidelines, and 3.4% according to their own information.

In terms of treatment, 85.0% (494/581) of respondents reported that they care for children and adolescents with ADHD in their practice and also write the prescriptions. A small proportion of respondents indicated that they either provide treatment without writing prescriptions (9.6%) or have no patients with ADHD in their practice at all (2.4%).

### Attitudes toward ADHD and medication treatment for ADHD

Bartlett’s test of sphericity (x^2^ (66) = 1515.48, *p* < .001) and Kaiser–Meyer–Olkin measure of sampling adequacy (KMO = .844) for the ADHD attitudes survey instrument indicated that the variables were suitable for factor analysis. Thus, a principal component analysis with varimax rotation was performed. Although this indicated the presence of four factors with eigenvalues greater than 1.0, based on the scree plot and theoretical considerations, a two-factor solution was chosen, which explained 43.2% of the variance. Table 2 shows the rotated factor loadings and Cronbach alpha coefficients for the two-factor model (positive and negative attitudes). Statements in factor 1 described “positive” attitudes (Generalized: acceptance of ADHD as a valid disease) and in factor 2 – “negative” attitudes (Generalized: rejection of ADHD as a valid disease). The internal consistency reliability was adequate with the Cronbach alpha values for the two factors.

The sum scores based on the factors were calculated for each respondent. Individual sum scores were compared and assigned to the following groups: those with more positive attitudes (higher individual scores in factor 1), those with more negative attitudes (higher scores in factor 2), and those with not clearly defined attitudes (equal scores for factor 1 and factor 2) toward ADHD. The vast majority of our respondents have a positive attitude toward ADHD, i.e., ADHD is a valid disease that should be treated. A small group, as many as 6.7%, disagrees with this opinion (Table 3). This complicates further evaluation as far as the regional correlation is concerned. The attempt to perform a correlation analysis of more negative attitudes with small-scale regionally low prevalence failed due to the low numbers. We then additionally looked at the four federal states in which there were several counties with very low prevalence (Hessen, Baden-Wurttemberg, Bavaria, Schleswig-Holstein). Here, too, the numbers were ultimately too small to allow conclusions to be drawn at a statistical level.

**Table 3:** Attitude of pediatricians toward ADHD in Germany

ADHD attitude	Study population n (*N* = 581)	Percent (%)
More negative	39	6.7
More positive	497	85.5
equally positive and negative	10	1.7
Missing values	35	6.0

Physicians with positive attitudes according to factor analysis were older and more often had a neuropediatric specialization. Attitudes were slightly more negative among younger physicians (Table 4).

**Table 4:** Multiple linear regression analysis of factors associated with positive and negative attitudes of pediatricians toward ADHD in Germany

Variables	Model 1: Factor 1 – positive attitudes *n* = 515 R^2^_adj_ = .114, F = 17.546, *p* = <.001				Model 2: Factor 2 – negative attitudes *n* = 517 R^2^_adj_ = .026, F = 4.411, *p* = .002			
	B	SE	β	p	B	SE	β	p
(Constant)	11.552	1.071		<.001	14.505	1.203		<.001
Age	.122	.022	.366	<.001	−.083	.025	−.230	.001
Sex (female/Ref. male)	226	.240	.040	.346	−.275	.271	−.045	.311
Neuropediatrician (yes/Ref. no)	1.501	.347	.183	<.001	−.701	.399	−.078	.079
Professional years as a pediatrician (≥21/Ref. ≤20)	−.635	.369	−.113	.086	.629	.415	.103	.130

Ref. = reference group

The seven statements assessing attitudes to medication treatment for ADHD were too heterogeneous and correlated very poorly with each other; therefore, the extraction and interpretation of factors “positive” and “negative” was not possible, which is also due to the construct of the questions (Fig. 1). The question was more focused on the settings themselves than designed to distinguish between “right” and “wrong” afterward.

## Discussion

The starting point of our considerations was the fact that medical decisions do not always depend on the available knowledge but also on attitudes. This is clearly demonstrated by the different vaccination rates against COVID-19 among medical personnel. However, other diseases also reflect such different attitudes. For years, there have been wide regional variations in the prevalence of ADHD in Germany. No plausible explanation has yet been found for the variations in ADHD prevalence and also methylphenidate prescription rates within Germany. For example, the effect of variables such as gender and age structure, social status of parents, availability of specialists, etc., on the regionally varying ADHD diagnosis rates has been analyzed. In some studies, a small influence of urbanity and specialist density is suspected, but this cannot be conclusively proven. Based on our results, we have no evidence that attitudes toward ADHD or medication among practicing pediatricians may play a role in the differences. On the other hand, our research does not allow us to exclude such a relationship with certainty, since the number of responses is too small. After all, 19.5% of respondents rather or strongly disagree that “ADHD is a clearly defined psychiatric disorder.” Even 27.2% disagree with the statement that “The etiology of ADHD lies in a predominantly genetically caused cerebral developmental disorder.”

The demographic data, distribution of responses by region, and other basic data reflect well the distribution of the professional association’s membership, although we do not wish to claim statistical representativeness. Response trends reflect current knowledge and guidelines. Of course, there are limitations regarding the validity due to the overall low response rate, although this is comparable to other studies. Fitzgerald sent more than 22,000 emails and postal invitations and 134 health professionals completed the survey (Fitzgerald and McNicholas 2014).

When comparing our data with other studies, it is important to keep in mind the different health care settings in each country. The setting with primary care pediatricians already exists in a number of other countries (e.g., Croatia, Finland, France, Italy, Portugal, Spain). However, this system does not exist in the countries where the main focus of the previous investigations was (UK, Australia, etc.). The different classification systems in the respective countries must also be taken into account, which can sometimes lead to different classifications (e.g., International Classification of Diseases (ICD-10), French Classification of Child and Adolescent Mental Disorders (CFTMEA) or Diagnostic and Statistical Manual of Mental Disorders (DSM-5)). Vallée also reported some cultural differences in training and conceptualization, particularly with regard to ADHD, which he then related to the very different prescription frequencies of stimulants in, for example, a comparison of France and the United States (Vallée 2011).

There are several studies in which surveys were conducted, especially among general practitioners, and their results differ significantly from ours. In one survey, 665 Iranian general practitioners cited sugar and food additives as the cause in 37% of cases, 52% chaotic and dysfunctional family, and 83% stated children with ADHD misbehave because they do not want to obey rules. Almost half of respondents believed that they had adequate knowledge about ADHD (Ghanizadeh and Zarei 2010). In a systematic narrative review, Tatlow-Golden concludes that general practitioners show mixed attitudes to the diagnosis itself and “and, crucially, very little interest at all in shared care” (Tatlow-Golden et al. 2016). Besides genetic and chemical influences, general practitioners in a UK based survey rated “quality of parenting” among the top three factors influencing ADHD (Salt et al. 2005).

Similar results can be seen in an Australian survey, albeit an older one, in which, for example, 77% of 399 general practitioners classified inadequate parenting, 75% ineffective discipline in the home, and 12% fast food as significant factors in the development of ADHD (Shaw et al. 2002). Even though there is no direct comparability due to the different composition of the respondents and the concepts have also changed somewhat over the years, the answers of the pediatricians in our survey can be classified as very pleasing and predominantly in accordance with national and international guidelines and current scientific concepts. Comparable results with a very similar approach can be seen in a survey in Ireland (Adamis et al. 2018). There, too, general practitioners were surveyed, and most response trends were in the same direction as in our study. The biggest difference was found in the statement “Parents seek ADHD diagnosis as an excuse for their child’s bad behavior,” which was answered with “yes” by 30.3% of the Irish GPs and which was only agreed with by 14.6% of our respondents.

As for the survey on attitudes toward medications and treatment, we also see very encouraging results in our study. This concerns, for example, agreement with stimulant treatment in accordance with the medical guidelines and predominately as part of multimodal therapy. The question about “sedation” by the drugs is also answered in the affirmative by only an absolute minority, whereas most respondents are likely to have focused on the mechanism of action with improved “filter function.”

We consider information about the attitudes of different groups - using the example of ADHD of physicians, psychologists, teachers and educators, parents and the affected persons themselves - to be important in order to better understand possible differences in diagnostics and therapy. Certain disorders may well be subject to stigmatization, as Goffmann already predicted for certain disorders in particular (Goffmann 1963; Mueller et al. 2012). This applies primarily to those diseases whose cause remains unknown or difficult to grasp and where there is also the opinion that the symptoms must be influenceable by the patients themselves. In the treatment of diseases, knowledge plays a role on the one hand but then also attitudes (Sciutto et al. 2016). The selection of the respective therapy procedures also depends to a not inconsiderable extent on attitudes. This concerns the parents and affected children but also the physicians.

### Limitations

The main limitation of our study is the low response rate. This means that regional differentiation is not possible, and the general validity is also limited. However, our response rate is in similar, but even rather higher, ranges than comparable studies with a high number of mailings (Fitzgerald and McNicholas 2014). Furthermore, we cannot rule out the possibility that the results were distorted by a selection of respondents. Possible influencing factors include coverage bias, non-response bias and self-selection bias. These are known to lead to insufficient data quality in voluntary online surveys (Bethlehem 2010). If the pediatricians motivated to participate differed systematically in their opinions from the entirety of pediatricians, then the overall significance could be distorted. Thus, it is at least conceivable that “ADHD opponents” participated less in the responses than those with a positive attitude, if opponents do not want to have anything to do with this topic. In addition, we lack information from child and adolescent psychiatrists, who also care for a certain number of ADHD patients in Germany. Nevertheless, we believe that our work makes some contribution to a better understanding also in international comparison, as it is, to the best of our knowledge, the first to investigate pediatricians’ attitudes toward ADHD for Germany. To further attempt to clarify prevalence differences, it might be helpful to conduct open qualitative or semi-structured interviews in different selected regions and thus compare areas with very high and very low prevalence. The prevalence figures are available and a high percentage of the respective colleagues in the regions would have to agree to participate. Data protection aspects must be taken into account and voluntariness must be ensured.

The use of factor analysis can be seen as a further limitation since this simplifies the assessment within the framework of a spectrum as a categorical division into two dimensions (“positive” and “negative”). Also, the wording used in this regard is not accurate in every case. Nevertheless, we have chosen this method because it allows a subsequent multivariate analysis and a comparability with other studies is given (Adamis et al. 2018).

## Conclusion

Even though the number of responses was not sufficient to take a closer look at regional differences, we still consider our results to be relevant. There is a satisfying agreement with the guidelines as far as the attitudes of the German pediatricians surveyed are concerned. We consider more far-reaching knowledge about attitudes to certain questions also among different groups - e.g., patients, relatives, teachers, physicians - to be important, since these might represent relevant explanations for conditions in medical care from the point of view of health care research.

## Supplementary information

Supplemental file 1 ADHD-Questionnaire-German-Original (PDF 213 kb)

Supplemental file 2 ADHD-Questionnaire-English (PDF 123 kb)

## Data Availability

The datasets generated during and/or analyzed during the current study are available from the corresponding author on reasonable request.

## Footnotes

1 In our opinion, the designations as “positive” and “negative” are somewhat unfortunate, as they imply a classification. We would rather prefer “in accordance with current guidelines” and “deviating from current guidelines,” (Arbeitsgemeinschaft der Wissenschaftlichen Medizinischen Fachgesellschaft (AWMF) 2017) but use the designations chosen so far because of the previous publications.
