# Supplementary material for: Is it all about knowledge? A survey of attitudes toward ADHD among German pediatricians
Source: Z Gesundh Wiss. 2022 Sep 23:1–9. Online ahead of print. doi: 10.1007/s10389-022-01758-4 (PMC9510220; doi:10.1007/s10389-022-01758-4)
Supplement: Supplementary file 1 — ADHD-Questionnaire-German-Original (PDF 213 kb) [file 10389_2022_1758_MOESM1_ESM.pdf]

EinstellungADHS → Eistellung_ADHS 04.01.2021, 12:12

Seite 01

SS

Willkommen zum Fragebogen!

Wir bedanken uns, dass Sie sich Zeit nehmen, den Fragebogen zu beantworten.

Die Umfrage erfolgt mit Unterstützung des Berufsverbandes der Kinder- und Jugendärzte (BVKJ e.V.) sowie der AG ADHS e.V.. Mit Hilfe dieser Befragung sollen die Einstellungen der Kinder- und Jugendärztinnen und -ärzte zu ADHS in Deutschland ermittelt werden.

Die Datenerhebung und -auswertung erfolgt im Rahmen einer Promotionsarbeit und sollen im Laufe des Jahres 2020 für eine Publikation aufbereitet werden.

Bitte nutzen Sie zur Navigation ausschließlich die Buttons **„Zurück“** und **„Weiter“** auf der jeweiligen Seite unten. Nutzen Sie bitte **auf keinen Fall den „Zurück“ Button** Ihres Browsers. Die Umfrage würde dadurch aus technischen Gründen abgebrochen.

Es ist uns bewusst, dass wir mit unseren Fragen nicht die gesamte Komplexität des Themas abbilden. Wir legen aber Wert darauf, dass die Umfrage in einigermaßen vertretbarer Zeit zu beantworten ist und werden auch die Interpretation der erhobenen Daten mit der gegebenen Sorgfalt durchführen. Teilweise greifen wir auf Formulierungen aus anderen Studien zurück, um eine Vergleichbarkeit zu gewährleisten.

Die Befragung wird ca. 5 Minuten in Anspruch nehmen.

Vielen Dank!

Marie Elise Fechner, Yuliya Mazheika, Prof. Dr. med. Peter Borusiak und Dr. med. Folkert Fehr

---

Seite 02

Zu Beginn zunächst noch einige Anmerkungen zur Wahrung Ihrer Anonymität und zum Datenschutz:

- Diese Umfrage wird von einem Forscherteam im Rahmen einer Promotionsarbeit durchgeführt und ausgewertet. Ihre Angaben werden nicht an unbefugte Dritte und lediglich als zusammengefasste Ergebnisse, aus denen keine Rückschlüsse auf Ihre individuelle Person gezogen werde können, an die Leitung des Projektes weitergegeben.
- Unsere Datenschutzhinweise können Sie unter folgendem Link herunterladen: Datenschutzhinweise

○ Ja, ich habe die Informationen zum Datenschutz gelesen, bin damit einverstanden und möchte teilnehmen.

○ Nein, ich möchte nicht an der Umfrage teilnehmen.

Seite 03

1. Welches Geschlecht haben Sie?

- ◯ weiblich
- ◯ männlich
- ◯ divers
- ◯ möchte ich nicht angeben

2. Wie alt sind Sie?

[ ] (Alter in Jahren)

Seite 04

3. Sind Sie Facharzt/in für Kinder- und Jugendmedizin?

- ◯ ja, weiter mit der **Frage 4**
- ◯ nein, weiter mit der **Frage 5**

4. Seit wie vielen Jahren sind Sie als Facharzt/in für Kinder- und Jugendmedizin tätig?

[Bitte auswählen]

5. Führen Sie die Schwerpunktbezeichnung Neuropädiatrie?

- ◯ ja
- ◯ nein

6. Haben Sie eine der folgenden Zusatzqualifikation?
Bitte wählen Sie eine der folgenden Antworten

- ◯ ja, als Facharzt/in für Kinder- und Jugendpsychiatrie und -Psychotherapie
- ◯ ja, als Kinder- und Jugendlichenpsychotherapeut/in
- ◯ ja, als Psychologe/in (Dipl. oder Master)
- ◯ ja, als ärztliche/r Psychotherapeut/in
- ◯ nein
- ◯ Sonstiges: [ ]

7. Kennen sie die Arbeitsgemeinschaft ADHS (AG ADHS)?

- ◯ nein
- ◯ ja, ich bin aber kein Mitglied
- ◯ ja, ich bin Mitglied der AG ADHS

Seite 05

8. In welchem Setting sind Sie vorwiegend in der Kinder-und Jugendmedizin tätig?

Bitte wählen Sie eine der folgenden Antworten

- ○ Einzelpraxis
- ○ Berufsausübungsgemeinschaft
- ○ Klinik
- ○ Sozialpädiatrisches Zentrum
- ○ Öffentlicher Gesundheitsdienst
- ○ Sonstiges: [ ]

9. Wo liegt der Ort Ihrer Erwerbstätigkeit?

Bitte geben Sie das Bundesland und die Postleitzahl Ihres Arbeitsortes an.

Bundesland [Bitte auswählen]

Wir benötigen die Postleitzahl um einen möglichen Zusammenhang mit den unterschiedlichen Prävalenzdaten von ADHS in Deutschland zu untersuchen. Wir wollen und werden keine persönliche Identifikation hierdurch herbeiführen. Wir bitten Sie, uns zu vertrauen und möglichst die Postleitzahl komplett anzugeben, mindestens aber die ersten 2 Ziffern.

Postleitzahl [ ]

Seite 06

10. Wie schätzen Sie die ADHS-Prävalenz in Deutschland ein?

Bitte wählen Sie eine der folgenden Antworten

- ○ ADHS wird **über**diagnostiziert
- ○ Die Diagnosestellung erfolgt bezogen auf die Prävalenz überwiegend korrekt
- ○ ADHS wird **unter**diagnostiziert
- ○ ADHS wird manchmal unter- und manchmal überdiagnostiziert
- ○ weiß ich nicht

11. Wie gestaltet sich die Diagnosestellung von Kindern mit ADHS bei Ihnen?

Bitte wählen Sie eine der folgenden Antworten

- ○ In meiner Praxis gibt es keine Kinder mit ADHS
- ○ Ich führe in meiner Praxis keine Diagnostik durch, übernehme aber Patienten mit diagnostizierter ADHS
- ○ Ich überweise aktiv bei entsprechendem Verdacht an einen Kooperationspartner (z.B. Schwerpunktpraxis, Kinder- und Jugendpsychiater, SPZ)
- ○ Ich führe die Diagnostik in meiner Praxis selber nach eigenen Maßgaben durch
- ○ Ich führe die Diagnostik in meiner Praxis selber entsprechend der Leitlinien durch

12. Wie gestaltet sich Ihre Behandlung von Kindern mit ADHS?

Bitte wählen Sie eine der folgenden Antworten

- ○ Ich behandele Kinder und Jugendliche mit ADHS in meiner alltäglichen Praxis gar nicht
- ○ Ich behandele Kinder und Jugendliche mit ADHS, stelle aber keine BTM-Rezepte aus
- ○ Ich behandele Kinder und Jugendliche mit ADHS, stelle BTM-Rezepte aus, wenn die Indikation von anderen Kooperationspartnern (z.B. spezialisierte/r Kinder- und Jugendarzt/in, Kinder- und Jugendpsychiater/in) gestellt wurde
- ○ Ich behandele Kinder und Jugendliche mit ADHS eigenständig bzw. im eigenen Team und stelle auch selber BTM-Rezepte aus
- ○ Sonstiges: ____________________

13. Hier geht es um Ihre <u>persönliche und subjektive Einstellung.</u> Bitte geben Sie an, inwieweit Sie den folgenden Aussagen zustimmen.

Zutreffendes bitte ankreuzen

	stimme **gar nicht zu**	stimme **eher nicht zu**	stimme **eher zu**	stimme **voll zu**
ADHS ist eine klar definierte Erkrankung	○	○	○	○
ADHS ist eine Modediagnose	○	○	○	○
ADHS ist eine gesellschaftlich akzeptierte Entschuldigung für schlechtes Verhalten	○	○	○	○
Eine ADHS Diagnose ist hilfreich für das Kind	○	○	○	○
Die Diagnose ADHS führt zur Stigmatisierung von Kindern	○	○	○	○
Kinder mit ADHS verhalten sich schlecht, weil sie sich nicht an die Regeln halten wollen	○	○	○	○
Eltern wünschen die Diagnose, um das schlechte Benehmen ihrer Kinder zu entschuldigen	○	○	○	○
Eine ADHS-Diagnose entlastet Familien vom Stress und unterstützt die Problemlösung	○	○	○	○
Die Ätiologie einer ADHS liegt in einer überwiegend genetisch verursachten zerebralen Entwicklungsstörung	○	○	○	○
Chaotische und dysfunktionale Familienstrukturen sind die Ursache für ADHS	○	○	○	○
ADHS beruht häufig auf einem Erziehungsfehler	○	○	○	○
ADHS kann durch eine Diät gebessert werden	○	○	○	○
Ich fühle mich sicher im Umgang mit Patienten mit ADHS	○	○	○	○

Seite 08

14. Hier geht es um Ihre rein persönliche Einstellung und nicht unbedingt um die Vorgaben der Leitlinien. Bitte geben Sie an, inwieweit Sie den folgenden Aussagen zum Thema „Medikamentöse Behandlung bei ADHS“ zustimmen.

Zutreffendes bitte ankreuzen

	stimme **gar nicht zu**	stimme **eher nicht zu**	stimme **eher zu**	stimme **voll zu**
Stimulanzien sind zur Behandlung von ADHS im Schulalter angemessen	○	○	○	○
Stimulanzien sollten mit äußerster Zurückhaltung und nur bei sehr schwer betroffenen Kindern eingesetzt werden	○	○	○	○
Stimulanzien sollten auch bei leichteren Fällen und nicht nur bei sehr schwer betroffenen Kindern eingesetzt werden	○	○	○	○
Stimulanzien sollten leitlinienkonform im Rahmen einer multimodalen Therapie eingesetzt werden	○	○	○	○
Die Kinder sollen durch Stimulantien ruhig gestellt werden	○	○	○	○
Ich habe in meiner Praxis viele Eltern, die Stimulantien für Ihre Kinder wollen, obwohl diese gar kein ADHS haben	○	○	○	○
Ich habe in meiner Praxis viele Eltern, die eine Medikation mit Stimulantien ablehnen, obwohl ich diese für indiziert halte	○	○	○	○

15. Bitte vervollständigen Sie die folgende Aussage mit entsprechenden Prozentangaben.

freie Zahl zwischen 0 und 100 eintragbar

Ich halte eine medikamentöse Behandlung von [ ] % der Patienten mit ADHS für angemessen.

Letzte Seite

Vielen Dank für Ihre Teilnahme!

Wir möchten uns ganz herzlich für Ihre Mithilfe bedanken.

Ihre Antworten wurden gespeichert, Sie können das Browser-Fenster nun schließen.

Marie Elise Fechner, Universität Witten Herdecke – 2020
