# Supplementary material for: Is it all about knowledge? A survey of attitudes toward ADHD among German pediatricians
Source: Z Gesundh Wiss. 2022 Sep 23:1–9. Online ahead of print. doi: 10.1007/s10389-022-01758-4 (PMC9510220; doi:10.1007/s10389-022-01758-4)
Supplement: Supplementary file 2 — ADHD-Questionnaire-English (PDF 123 kb) [file 10389_2022_1758_MOESM2_ESM.pdf]

Welcome to the questionnaire!

Thank you for taking the time to answer the questionnaire.

The survey is carried out with the support of the Berufsverband der Kinder- und Jugendärzte (BVKJ e.V.) and the AG ADHS e.V. The purpose of this survey is to determine the attitudes of pediatricians to ADHD in Germany.

The data collection and analysis will be carried out as part of a doctoral thesis and will be prepared for publication in the course of 2020.

Please use only the **"Back"** and **"Next"** buttons at the bottom of each page for navigation. Please **do not use the "Back" button of your browser** under any circumstances. This would cancel the survey for technical reasons.

We are aware that our questions do not cover the entire complexity of the topic. However, we attach importance to the fact that the survey can be answered in a reasonably reasonable amount of time, and we will also interpret the data collected with due care. In some cases, we use formulations from other studies to ensure comparability.

The survey will take approximately 5 minutes to complete.

Thank you.
Marie Elise Fechner, Yuliya Mazheika, Prof. Dr. med. Peter Borusiak and Dr. med. Folkert Fehr

---

To begin with, a few more remarks about preserving your anonymity and data protection:

- This survey is being conducted and evaluated by a team of researchers as part of a doctoral thesis Your data will not be passed on to unauthorized third parties and only as summarized results, from which no conclusions can be drawn about your individual person, to the management of the project.
- You can download our privacy policy under the following link: Data protection information

  □ Yes, I have read the information on data protection, agree to it and would like to participate.
  □ No, I do not wish to participate in the survey.

---

1. **Please specify your gender?**
    - □ female
    - □ male
    - □ diverse
    - □ I do not wish to specify

2. **How old are you?**
    ______________ (Age in years)

3. **Are you a pediatrician?**
    - □ Yes (continue with question 4)
    - □ No (continue with question 5)

4. **How long have you been working as pediatrician?**
    - □ < 5 years
    - □ 6-10 years
    - □ 11-15 years
    - □ 16-20 years
    - □ 21-25 years
    - □ 26-30 years
    - □ 31-35 years
    - □ 36-40 years
    - □ 40 years

5. **Do you have a further formal qualification as a pediatric neurologist?**
    - □ Yes
    - □ No

6. **Do you have any of the following additional qualifications?**
    Please choose one of the following answers

    - □ Yes, as a specialist in child and adolescent psychiatry and psychotherapy
    - □ Yes, as a child and adolescent psychotherapist
    - □ Yes, as a psychologist (Dipl. or M. Sc.)
    - □ Yes, as a medical psychotherapist
    - □ No
    - □ Other_____________

7. **Do you know the ADHD working group (AG ADHD)?**
    - □ No
    - □ Yes, but I am not a member
    - □ Yes, I am a member of the AG ADHD

8. In which setting do you predominantly work?
Please choose one of the following answers

- □ Private practice (single)
- □ Private practice (group)
- □ Hospital
- □ Social pediatric center
- □ Public health service
- □ Other_____________

9. Where is the location of your employment?
Please enter the state and zip code of your work location.

German federal state _____________

We need the zip code to investigate a possible correlation with the different prevalence data of ADHD in Germany. We do not want to and will not use it for personal identification. We ask you to trust us and to provide the complete postal code if possible, but at least the first 2 digits.

Postalcode _______

10. What do you think about the prevalence of ADHD in Germany?
Please choose one of the following answers

- □ ADHD is overdiagnosed
- □ The diagnosis is mostly correct in relation to the prevalence
- □ ADHD is underdiagnosed
- □ ADHD is sometimes underdiagnosed and sometimes overdiagnosed
- □ no answer

11. How do you diagnose children with ADHD?
Please choose one of the following answers

- □ In my practice there are no children with ADHD
- □ I do not perform diagnostics in my practice, but I accept patients diagnosed with ADHD
- □ I actively refer patients with suspected ADHD to a cooperation partner (e.g. Child and adolescent psychiatrist, Social pediatric center)
- □ I carry out the diagnostics myself in my practice according to my own specifications
- □ I carry out the diagnostics in my practice myself according to the medical guidelines

12. How do you treat children with ADHD?

Please choose one of the following answers

- ◻ I do not treat children and adolescents with ADHD at all
- ◻ I treat children and adolescents with ADHD, but I do not write prescriptions for stimulants
- ◻ I treat children and adolescents with ADHD and write prescriptions for stimulants if the indication was made by one of the cooperation partners
- ◻ I treat children and adolescents with ADHD and also write prescriptions for stimulants myself
- ◻ Other_______________

13. This is about your <u>personal and subjective attitude</u>. Please indicate the extent to which you agree with the following statements.

Please mark with a cross

	**Strongly disagree**	**Rather disagree**	**Somewhat agree**	**Strongly agree**
ADHD is a clearly defined psychiatric disorder	O	O	O	O
ADHD is a new, 'fashionable' disorder	O	O	O	O
ADHD is society's excuse for badly behaved children	O	O	O	O
An ADHD diagnosis is helpful for a child	O	O	O	O
An ADHD diagnosis is stigmatizing for a child	O	O	O	O
Children with ADHD misbehave because they do not want to follow the rules	O	O	O	O
Parents seek ADHD diagnosis as an excuse for their child's bad behaviour	O	O	O	O
An ADHD diagnosis relieves families from stress and supports problem-solving	O	O	O	O
The etiology of ADHD lies in a predominantly genetically caused cerebral developmental disorder	O	O	O	O
Chaotic and dysfunctional family is the etiology of ADHD	O	O	O	O
ADHD can be caused by poor parenting practices	O	O	O	O
ADHD can be improved by diet	O	O	O	O
I feel confident in dealing with patients with ADHD	O	O	O	O

14. This question is purely about your personal attitude and not necessarily about what the guidelines say. Please indicate to what extent you agree with the following statements on the topic of "Medication treatment for ADHD".

Please mark with a cross

	**Strongly disagree**	**Rather disagree**	**Somewhat agree**	**Strongly agree**
Stimulants are appropriate for the ADHD treatment of school-aged children	O	O	O	O
Stimulants should be used with extreme caution and only in children with very severe ADHD	O	O	O	O
Stimulants should also be used in milder cases and not only in very severely affected children	O	O	O	O
Stimulants should be used in accordance with guidelines as part of multimodal therapy	O	O	O	O
The children are to be sedated by stimulants	O	O	O	O
I have many parents in my practice who want stimulants for their children even though they do not have ADHD at all	O	O	O	O
I have many parents in my practice who refuse to medicate with stimulants even though I think they are indicated	O	O	O	O

15. Please complete the following statement with appropriate percentages.

Numbers between 0 and 100 can be entered

I consider a drug treatment of _____% of patients with ADHD to be appropriate.

Thank you for your participation!

We would like to thank you very much for your assistance.

Your answers have been saved, you can now close the browser window.
